# Supplementary material for: Structure of the decoy module of human glycoprotein 2 and uromodulin and its interaction with bacterial adhesin FimH
Source: Nat Struct Mol Biol. 2022 Mar 10;29(3):190–3. doi: 10.1038/s41594-022-00729-3 (PMC8930769; doi:10.1038/s41594-022-00729-3)
Supplement: Supplementary file 1 — Supplementary Tables 1–3 and references. [file 41594_2022_729_MOESM1_ESM.pdf]

nature portfolio

https://doi.org/10.1038/s41594-022-00729-3

Supplementary information

Structure of the decoy module of human glycoprotein 2 and uromodulin and its interaction with bacterial adhesin FimH

In the format provided by the authors and unedited

Supplementary Table 1
X-ray data collection, refinement and validation statistics

	**GP2 decoy module crystal form I (*P*1)** (PDB 7P6R)	**GP2 decoy module crystal form II (*P*$2_12_12_1$)** (PDB 7P6S)	**GP2 decoy module crystal form III (*C*2)** (PDB 7P6T)
**Data collection**			
Space group	*P*1 [1]	*P*$2_12_12_1$ [19]	*C*2 [5]
Cell dimensions			
a, *b*, *c* (Å)	33.04, 46.53, 57.44	33.48, 59.50, 87.04	90.15, 33.66, 59.63
α, *β*, γ (°)	68.750, 75.873, 72.398	90, 90, 90	90, 111.839, 90
Resolution (Å)	52.9–1.90 (1.97–1.90)*	49.1–1.35 (1.39–1.35)	29.0–1.40 (1.47–1.40)
No. unique reflections	22914 (2161)	38991 (2960)	32721 (4653)
Completeness (%)	96.6 (91.4)	99.7 (98.2)	98.4 (96.8)
Redundancy	3.5 (3.2)	6.0 (4.7)	6.9 (6.7)
R_{merge}	0.099 (0.509)	0.153 (2.318)	0.104 (3.379)
R_{meas}	0.118 (0.611)	0.167 (2.612)	0.112 (3.663)
R_{pim}	0.063 (0.333)	0.067 (1.180)	0.042 (1.396)
Wilson B-factor (Å^2)	20.1	13.2	18.9
I / σI	8.8 (2.6)	7.0 (0.7)	8.6 (0.6)
$CC_{1/2}$	0.99 (0.89)	1.00 (0.40)	1.00 (0.48)
CC*	1.00 (0.97)	1.00 (0.75)	1.00 (0.81)
**Refinement**			
Resolution (Å)	52.9–1.90 (1.97–1.90)	49.1–1.35 (1.39–1.35)	29.0–1.40 (1.47–1.40)
No. reflections	22860 (2157)	38932 (2921)	32522 (4534)
No. free reflections	1579 (152)	2027 (153)	2018 (280)
R_{work}	0.233 (0.275)	0.194 (0.422)	0.194 (0.514)
R_{free}	0.280 (0.313)	0.224 (0.429)	0.223 (0.518)
No. non-H atoms	2400	1389	1288
Protein	2061	1122	1067
Ligand/ion	106	35	87
Water	233	232	134
No. protein residues	265	142	138
B-factors	28.7	18.5	31.3
Protein	27.9	16.5	28.6
Ligand/ion	38.7	18.2	53.4
Water	31.2	28.2	38.4
R.m.s. deviations			
Bond lengths (Å)	0.007	0.004	0.003
Bond angles (°)	0.73	0.72	0.67
Validation			
MolProbity score	1.23	0.95	0.66
Clashscore	4.59	1.83	0.45
Rotamer outliers (%)	0.0	0.0	0.0
Ramachandran plot			
Overall Z-score	-1.29 ± 0.44	-1.47 ± 0.55	-1.12 ± 0.60
Favored (%)	98.4	98.6	98.5
Allowed (%)	1.6	1.4	1.5
Disallowed (%)	0.0	0.0	0.0

* Values in parentheses are for highest-resolution shell

Supplementary Table 2
Pathogenic UMOD D10C domain missense mutations

UMOD mutation	Equivalent GP2 residue*	Predicted mutation effect based on structural information	Disease reported[§]	Reference
D172H	**D61**	Affects the relative orientation of the β-hairpin and D10C domain by disrupting the salt bridge between D172 and K265 (K155 in GP2)	TN	66
P173L<br>P173R	**P62**	The mutated residue clashes against invariant W202 (W92 in GP2), affecting the interface between the D10C domain and the β-hairpin	UAKD<br>FJHN	4<br>67
C174R	**$C_1$63**	Destroys conserved disulfide bond C_1-C_8	UAKD	68
R185C<br>R185G<br>R185H<br>R185L<br>R185S	**R74**	Disrupts the interaction between helix 3_{10}B and loop 3_{10}B-βB	TN<br>FJHN<br>TN<br>ADTKD<br>FJHN	66<br>69<br>66<br>70<br>71
C195F<br>C195Y	**$C_2$85**	Destroys conserved disulfide bond C_2-C_9	FJHN<br>FJHN	72<br>73
D196N<br>D196Y	**D86**	Disrupts the interaction between loop 3_{10}B-βB and helix 3_{10}B	FJHN<br>FJHN	69<br>74
W202C<br>W202S	**W92**	Disrupts the interaction between the D10C domain and the β-hairpin	UAKD<br>FJHN	4<br>72
R204G<br>R204P	**R94**	Disrupts the cation-π interaction with βG Y271 (Y161 in GP2) and affects the interface between the 3_{10}A-βA region and the D10C domain β-strand core	FJHN<br>TN	71<br>66
G210D<br>G210S	G100	The mutated residue clashes against the C-terminal end of D10C that includes β-strand I	UAKD<br>TN	4<br>66
R212C	R102	Interferes with correct disulfide bond formation	UAKD	75
C217G<br>C217R<br>C217W	$C_3$107	Destroys conserved disulfide bond C_3-C_6	FJHN, TN<br>FJHN<br>FJHN	66,71<br>76<br>69
C223R<br>C223Y	**$C_4$113**	Destroys conserved disulfide bond C_4-C_{10}	FJHN<br>FJHN	69<br>77
T225K<br>T225M	T115	Disrupts hydrogen bonding between the Thr hydroxyl group and main chain atoms; introduces clashes with β-strands D/H	MCKD2<br>FJHN	78<br>71
M229R	M119	Disrupts D10C hydrophobic core	FJHN/MCKD	79
W230R	W120	Disrupts a key D10C residue whose aromatic side chain lies between the C_3-C_6 and C_5-C_7 disulfides	UAKD	80
P236L<br>P236Q<br>P236R<br>P236S	P126	Disrupts the interaction between loop βD-βE and the D10C domain β-strand core	FJHN<br>FJHN<br>FJHN<br>UAKD	72<br>81<br>82<br>4
C248S<br>C248W	$C_5$138	Destroys conserved disulfide bond C_5-C_7	UAKD<br>MCKD2	80<br>78
H250L<br>H250Q	H140	Disrupts the packing of the His ring against the C_3-C_6 disulfide (on the opposite side of W230 (GP2 W120)	TN<br>TN	66<br>83
C255Y	$C_6$145	Destroys conserved disulfide bond C_3-C_6	FJHN	84
C256G<br>C256Y	$C_7$146	Destroys conserved disulfide bond C_5-C_7	FJHN<br>UAKD	85<br>75
C267F	**$C_8$157**	Destroys conserved disulfide bond C_1-C_8	MCKD2, FJHN	86
G269C	G159	Interferes with correct disulfide bond formation and the β-turn between strands βF and βG	UAKD	4
G270C	G160	Interferes with correct disulfide bond formation and the β-turn between strands βF and βG	UAKD	87
V273F<br>V273L	**V163**	Introduces clashes into the hydrophobic core	FJHN/MCKD<br>TN	79<br>66
Y274C<br>Y274H	**Y164**	Destabilizes the structure of the βG strand, carrying the UMOD high-mannose glycan and, in the case of Y274C, may also interfere with correct disulfide bond formation	UAKD	80
C282R<br>C282S	$C_9$172	Destroys conserved disulfide bond C_2-C_9	FJHN<br>UAKD	71<br>4
L284P	L174	Affects closely located disulfide bond C_2-C_9	UAKD	4
C287F	**C_{10}177**	Destroys conserved disulfide bond C_4-C_{10}	ADTKD	88

* Residues shown in Fig. 1c-g and Extended Data Fig. 5c-g are highlighted in bold
[§] ADTKD, Autosomal Dominant Tubulointerstitial Kidney Disease; FJHN, Familial Juvenile Hyperuricemic Nephropathy; MCKD, Medullary Cystic Kidney Disease; TN, Tubulointerstitial Nephritis; UAKD, Uromodulin-Associated Kidney Disease

Supplementary Table 3
Cryo-EM data collection, refinement and validation statistics

	Full-length UMOD (EMD-10553 + EMD-13378) (PDB 7PFP)		UMOD branch + EGF IV/ FimH$_L$ complex (EMD-13794) (PDB 7Q3N)
**Data collection and processing**			
Magnification	130,000x		105,000x
Voltage (kV)	300		300
Electron exposure (e–/Å^2)	39.6		40
Defocus range (μm)	-1.5 to -3.5		-1 to -3
Pixel size (Å)	1.06		0.84
Body	Filament core + D10C domain	Branch	
Symmetry imposed	Helical (with 62.5 Å rise, 180.0° twist)	Non-helical	Helical (initial; with 65.2 Å rise, 180.0° twist); non-helical (final)
Initial particle images (no.)	412,322	412,322	3,767,790
Final particle images (no.)	288,403	114,206	225,819
Map resolution (Å)	3.35	6.1	7.4
FSC threshold	0.143	0.143	0.143
Map resolution range (Å)	3.0–4.2	5.0-6.8	6.4–7.9
**Refinement**			
Initial models used (PDB codes)	PDB 6TQK, AlphaFold2 model, PDB 7P6R/7P6S/7P6T		PDB 7PFP, PDB 6GTW
Model resolution (Å)			
masked	4.1		8.3
unmasked	4.4		8.5
FSC threshold	0.143		0.143
Map sharpening *B* factor (Å^2)	-200		-150
Model composition			
Non-hydrogen atoms	9,582		3,599
Protein residues	1,127		451
Carbohydrate residues	84		20
B factors (Å^2)			
Protein	315		404
Carbohydrate residues	406		291
R.m.s. deviations			
Bond lengths (Å)	0.005		0.003
Bond angles (°)	0.845		0.672
Validation			
MolProbity score	1.83		1.71
Clashscore	4.08		9.47
Poor rotamers (%)	2.4		0.5
Ramachandran plot			
Overall Z-score	-1.66 ± 0.24		-0.74 ± 0.37
Favored (%)	94.9		96.6
Allowed (%)	5.1		3.4
Disallowed (%)	0.0		0.0

Supplementary References

66. Bollée, G. *et al.* Phenotype and outcome in hereditary tubulointerstitial nephritis secondary to *UMOD* mutations. *Clin. J. Am. Soc. Nephrol.* **6**, 2429–2438 (2011).
67. Iguchi, A. *et al.* A novel mutation in the uromodulin gene in a Japanese family with a mild phenotype of familial juvenile hyperuricemic nephropathy. *CEN Case Rep.* **2**, 228–233 (2013).
68. Moskowitz, J. L. *et al.* Association between genotype and phenotype in uromodulin-associated kidney disease. *Clin. J. Am. Soc. Nephrol.* **8**, 1349–1357 (2013).
69. Williams, S. E. *et al.* Uromodulin mutations causing familial juvenile hyperuricaemic nephropathy lead to protein maturation defects and retention in the endoplasmic reticulum. *Hum. Mol. Genet.* **18**, 2963–2974 (2009).
70. Zhang, L.-L. *et al.* Autosomal dominant tubulointerstitial kidney disease with a novel heterozygous missense mutation in the uromodulin gene: A case report. *World J. Clin. Cases* **9**, 10249–10256 (2021).
71. Dahan, K. *et al.* A cluster of mutations in the UMOD gene causes familial juvenile hyperuricemic nephropathy with abnormal expression of uromodulin. *J. Am. Soc. Nephrol.* **14**, 2883–2893 (2003).
72. Kudo, E. *et al.* Familial juvenile hyperuricemic nephropathy: Detection of mutations in the uromodulin gene in five Japanese families. *Kidney Int*. **65**, 1589–1597 (2004).
73. Spain, H., Plumb, T. & Mikuls, T. R. Gout as a manifestation of familial juvenile hyperuricemic nephropathy. *J. Clin. Rheumatol.* **20**, 442–444 (2014).
74. Lhotta, K. *et al.* Familial juvenile hyperuricemic nephropathy: report on a new mutation and a pregnancy. *Clin. Nephrol.* **71**, 80–83 (2009).
75. Schaeffer, C. *et al.* Urinary secretion and extracellular aggregation of mutant uromodulin isoforms. *Kidney Int.* **81**, 769–778 (2012).
76. Hart, T. C. *et al.* Mutations of the *UMOD* gene are responsible for medullary cystic kidney disease 2 and familial juvenile hyperuricaemic nephropathy. *J. Med. Genet.* **39**, 882–892 (2002).
77. Bleyer, A. J., Trachtman, H., Sandhu, J., Gorry, M. C. & Hart, T. C. Renal manifestations of a mutation in the uromodulin (Tamm Horsfall protein) gene. *Am. J. Kidney Dis.* **42**, E20–E26 (2003).

78. Wolf, M. T. F. *et al.* Mutations of the *Uromodulin* gene in MCKD type 2 patients cluster in exon 4, which encodes three EGF-like domains. *Kidney Int.* **64**, 1580–1587 (2003).
79. Vylet'al, P. *et al.* Alterations of uromodulin biology: a common denominator of the genetically heterogeneous FJHN/MCKD syndrome. *Kidney Int.* **70**, 1155–1169 (2006).
80. Zaucke, F. *et al.* Uromodulin is expressed in renal primary cilia and *UMOD* mutations result in decreased ciliary uromodulin expression. *Hum. Mol. Genet.* **19**, 1985–1997 (2010).
81. Liu, M. *et al.* Novel *UMOD* mutations in familial juvenile hyperuricemic nephropathy lead to abnormal uromodulin intracellular trafficking. *Gene* **531**, 363–369 (2013).
82. Bernascone, I. *et al.* Defective intracellular trafficking of uromodulin mutant isoforms. *Traffic* **7**, 1567–1579 (2006).
83. Raffler, G., Zitt, E., Sprenger-Mähr, H., Nagel, M. & Lhotta, K. Autosomal dominant tubulointerstitial kidney disease caused by uromodulin mutations: seek and you will find. *Wien. Klin. Wochenschr.* **128**, 291–294 (2016).
84. Turner, J. J. O. *et al.* UROMODULIN mutations cause familial juvenile hyperuricemic nephropathy. *J. Clin. Endocrinol. Metab.* **88**, 1398–1401 (2003).
85. Takemasa, Y. *et al.* Familial juvenile hyperuricemia in early childhood in a boy with a novel gene mutation. *CEN Case Rep* **10**, 426–430 (2021).
86. Christiansen, R. E. *et al.* A mother and daughter with unexplained renal failure. *Nephron Clin. Pract.* **119**, c1–c9 (2011).
87. Nasr, S. H., Lucia, J. P., Galgano, S. J., Markowitz, G. S. & D'Agati, V. D. Uromodulin storage disease. *Kidney Int.* **73**, 971–976 (2008).
88. Gong, K. *et al.* Autosomal dominant tubulointerstitial kidney disease genotype and phenotype correlation in a Chinese cohort. *Sci. Rep.* **11**, 3615 (2021).
