# Supplementary material for: Structure of the decoy module of human glycoprotein 2 and uromodulin and its interaction with bacterial adhesin FimH
Source: Nat Struct Mol Biol. 2022 Mar 10;29(3):190–3. doi: 10.1038/s41594-022-00729-3 (PMC8930769; doi:10.1038/s41594-022-00729-3)
Supplement: Supplementary file 2 — Reporting Summary [file 41594_2022_729_MOESM2_ESM.pdf]

nature portfolio

Corresponding author(s): Luca Jovine

Last updated by author(s): Jan 31, 2022

# Reporting Summary

Nature Portfolio wishes to improve the reproducibility of the work that we publish. This form provides structure for consistency and transparency in reporting. For further information on Nature Portfolio policies, see our Editorial Policies and the Editorial Policy Checklist.

## Statistics

For all statistical analyses, confirm that the following items are present in the figure legend, table legend, main text, or Methods section.

| n/a | Confirmed | |
|---|---|---|
| ☐ | ☒ | The exact sample size (*n*) for each experimental group/condition, given as a discrete number and unit of measurement |
| ☐ | ☒ | A statement on whether measurements were taken from distinct samples or whether the same sample was measured repeatedly |
| ☒ | ☐ | The statistical test(s) used AND whether they are one- or two-sided<br>*Only common tests should be described solely by name; describe more complex techniques in the Methods section.* |
| ☒ | ☐ | A description of all covariates tested |
| ☒ | ☐ | A description of any assumptions or corrections, such as tests of normality and adjustment for multiple comparisons |
| ☒ | ☐ | A full description of the statistical parameters including central tendency (e.g. means) or other basic estimates (e.g. regression coefficient) AND variation (e.g. standard deviation) or associated estimates of uncertainty (e.g. confidence intervals) |
| ☒ | ☐ | For null hypothesis testing, the test statistic (e.g. *F*, *t*, *r*) with confidence intervals, effect sizes, degrees of freedom and *P* value noted<br>*Give P values as exact values whenever suitable.* |
| ☒ | ☐ | For Bayesian analysis, information on the choice of priors and Markov chain Monte Carlo settings |
| ☒ | ☐ | For hierarchical and complex designs, identification of the appropriate level for tests and full reporting of outcomes |
| ☒ | ☐ | Estimates of effect sizes (e.g. Cohen's *d*, Pearson's *r*), indicating how they were calculated |

*Our web collection on statistics for biologists contains articles on many of the points above.*

## Software and code

Policy information about availability of computer code

| | |
|---|---|
| Data collection | EPU 2.11.1, MXCuBE3 3.0 |
| Data analysis | AlphaFold Monomer 2.0; AS2TS server 09/2019; ByonicTM 3.4.0; ConSurf 2016; Coot 0.8.9.2-0.9.6; Cryo-EM-filament-picker 1.0; cryoSPARC 3.2; CTFFIND 4.1.14; Dali 5; ESPript 3.0; ISOLDE 1.2; MAFFT 7; MODELLER 10.1; MolProbity 4.5.1; MView 1.68; Namdinator 2.0; Phaser 2.8.3; PHENIX (phenix.autobuild, phenix.refine, phenix.table_one) 1.19.2_4158, dev_4282; Privateer MKIII-MKIV; PyMOL 2.4.2; RELION 3.0.8; Scipion 3.0.9; STRIDE 1.0; TEMPy:DiffMap 2; UCSF Chimera 1.11-1.15; UCSF ChimeraX 1.1-1.2; XDS Feb 5, 2021 BUILT=20210322; YASARA Structure 21.4.22 |

For manuscripts utilizing custom algorithms or software that are central to the research but not yet described in published literature, software must be made available to editors and reviewers. We strongly encourage code deposition in a community repository (e.g. GitHub). See the Nature Portfolio guidelines for submitting code & software for further information.

## Data

Policy information about availability of data

All manuscripts must include a data availability statement. This statement should provide the following information, where applicable:

- Accession codes, unique identifiers, or web links for publicly available datasets
- A description of any restrictions on data availability
- For clinical datasets or third party data, please ensure that the statement adheres to our policy

The UniProt (https://www.uniprot.org/) IDs for hGP2 and hUMOD are P55259 and P07911, respectively; the IDs of other sequences reported in the alignment of Extended Data Fig. 1b are Q9D733 (mGP2), Q91X17 (mUMOD), Q8WWZ8 (hLZP), Q8R4V5 (mLZP), Q8N2E2 (hVWDE) and Q6DFV8 (mVWDE). The Electron Microscopy Data Bank (EMDB; https://www.ebi.ac.uk/emdb/) ID of the UMOD filament map used for assembling the composite map shown in this work is

EMD-10553; the UMOD filament core and FimHL/trimannose coordinates used as starting models can be retrieved from the Protein Data Bank (PDB; http://www.rcsb.org) with IDs 6TQK and 6GTW, respectively.
Structure factors and atomic models for the P1, P212121 and C2 crystal forms of the GP2 decoy domain have been deposited in the PDB with accession codes 7P6R, 7P6S and 7P6T, respectively. Cryo-EM density maps of full-length UMOD and the UMOD branch + EGF IV/FimHL complex have been deposited in the EMDB with accession codes EMD-13378 and EMD-13794, respectively; the corresponding coordinates have been deposited in the PDB with accession codes 7PFP and 7Q3N.

# Field-specific reporting

Please select the one below that is the best fit for your research. If you are not sure, read the appropriate sections before making your selection.

☒ Life sciences ☐ Behavioural & social sciences ☐ Ecological, evolutionary & environmental sciences

For a reference copy of the document with all sections, see nature.com/documents/nr-reporting-summary-flat.pdf

# Life sciences study design

All studies must disclose on these points even when the disclosure is negative.

**Sample size**

No statistical methods were used to predetermine sample size.
For structure determination of the GP2 decoy module by X-ray crystallography, we measured diffraction from specimens that belonged to three different crystal forms (P1, P2(1)2(1)2(1) and C2). Using crystals harvested from multiple crystallization drops, we screened 119 samples and collected 13 P1, 15 P2(1)2(1)2(1) and 3 C2 datasets. The datasets belonging to each space group were then ranked by resolution and quality (based on the statistical indicators reported in Supplementary Table 1), and the best ones (which were processed to resolutions of 1.9 Å, 1.35 Å and 1.4 Å, respectively; Supplementary Table 1) were used for structure solution by molecular replacement and refinement.
For cryo-EM analysis of full-length UMOD and the UMOD branch/FimH(L) complex, we screened >20 grids of each sample at 0.8-1.8 mg mL-1 concentrations. The datasets used for structure determination consisted of 2,300 and 13,616 raw micrographs, respectively, from which 412,322 and 3,767,790 filaments were picked and used for 2D classification. The number of particles used in the final reconstructions was 288,403 (UMOD filament core + D10C domain), 114,206 (UMOD branch) and 225,819 (UMOD branch/FimH(L)) (Supplementary Table 3). This was sufficient to assemble a composite map of full-length UMOD with a nominal resolution of 6.1 Å, and to obtain a map of the UMOD branch/FimH(L) complex with a nominal resolution of 7.4 Å.
For biochemical experiments, we used amounts and concentrations of proteins that provided sufficient signal-to-noise ratios to obtain unambiguous results, based on previous knowledge of the corresponding experimental setups.

**Data exclusions**

As in the case of all single-crystal X-ray diffraction experiments, two high resolution choices were made for each dataset that could have at least potentially excluded part of the weakest reflections: first, a crystal-to-detector distance was chosen, based on an initial resolution estimate made by the beamline data collection/processing software; second, a more accurate high-resolution cutoff was chosen, based on the mean I/σI and CC(1/2) values obtained upon manual processing of the datasets. The latter choice was made following the established criteria described in PMIDs 23793146 and 26209821 (Methods-associated references 29 and 30).
Processing of the cryo-EM data for full-length UMOD has already been described in PMID 33196145 (reference 6 of the manuscript). For determining the structure of the UMOD branch/FimH(L) complex by cryo-EM, we only processed micrographs with an estimated resolution better than 8 A. As also detailed in the Methods, subsequent particle exclusions were performed at three different stages: (1) starting from a total of 13,616 raw micrographs, 3,767,790 helical segments were auto-picked and extracted on the basis of motion correction and CTF estimation; (2) based on 2D classification quality evaluated with cryoSPARC, a subset of 1,139,808 particles was then selected for further processing; and (3) because FimH(L) occupancy varied among filaments, segments with higher FimH(L) occupancy were selected during iterative RELION 3D classification runs, resulting in 225,819 homogeneous particles that were subjected to auto-refinement and postprocessing.
Finally, no data was excluded in conjunction with the biochemical experiments described in this manuscript.

**Replication**

Although the structures of the three crystal forms of the GP2 decoy module were obtained from diffraction data collected from single crystals (as commonly done in X-ray crystallography), as detailed in the section "Sample size" several specimens were screened and measured for each of them. For each crystal form, all of these samples were consistent in terms of morphology, space group and unit cell dimensions. Most importantly, the structures of the three different crystal forms of the protein are essentially equivalent (average Cα RMSD 0.6 A).
Cryo-EM single particle analysis averages independent particle observations, and – as reported in Supplementary Table 3 – 288,403 and 225,819 particles were averaged to yield the final 3D reconstructions of full-length UMOD and the UMOD branch/FimH(L) complex, respectively.
Biochemical experiments were successfully reproduced as detailed in the respective figure legends. Specifically, n=3 for the experiments shown in Fig. 1b, Fig. 2c, Extended Data Fig. 2b (GP2 decoy module, UMOD decoy module), Extended Data Fig. 2c and n=2 for the experiments of Extended Data Fig. 2a, Extended Data Fig. 2b (GP2 decoy module/FimH(L), UMOD decoy module/FimH(L)) and Extended Data Fig. 7b (GP2 decoy module/FimH(L), GP2 decoy module N65A/FimH(L)).

**Randomization**

X-ray crystallography: random assignment of reflections to working or free sets was automatically performed by PHENIX (P1 data) or XDS (P2(1)2(1)2(1) and C2 data).
Cryo-EM: The vitrified UMOD filaments (free or bound to FimH) used for structure determination by cryo-EM adopt random orientations on the XY plane of the EM grids, although – as previously described in PMID 33196145/reference 6 of the manuscript – they are significantly less randomly distributed along Z due to the fact that they tend to lie flat on the grids themselves. Assignment of particles into random half datasets was automatically performed by RELION during 3D reconstruction.
Biochemical experiments: these experiments did not involve or require randomization.

**Blinding**

Blinding was not applicable to the type of data that was analyzed in this study. In particular, knowledge of the identity of the molecules under investigation was required to express them, purify them and determine their structure, because the success of all these procedures depends on information (primary sequence, post-translational modifications etc.) that is specific to each experimental sample.

# Reporting for specific materials, systems and methods

We require information from authors about some types of materials, experimental systems and methods used in many studies. Here, indicate whether each material, system or method listed is relevant to your study. If you are not sure if a list item applies to your research, read the appropriate section before selecting a response.

## Materials & experimental systems

| n/a | Involved in the study |
|---|---|
| ☐ | ☒ Antibodies |
| ☐ | ☒ Eukaryotic cell lines |
| ☒ | ☐ Palaeontology and archaeology |
| ☒ | ☐ Animals and other organisms |
| ☐ | ☒ Human research participants |
| ☒ | ☐ Clinical data |
| ☒ | ☐ Dual use research of concern |

## Methods

| n/a | Involved in the study |
|---|---|
| ☒ | ☐ ChIP-seq |
| ☒ | ☐ Flow cytometry |
| ☒ | ☐ MRI-based neuroimaging |

## Antibodies

| | |
|---|---|
| Antibodies used | Primary antibody: Penta-His Antibody, BSA-free (QIAGEN, Cat. No. 34660, Lot 157046697).<br>Secondary antibody: Goat anti-Mouse IgG Fc Secondary Antibody, HRP (Invitrogen, Cat. No. A16084, Lot 62-47-012318). |
| Validation | The QIAGEN Penta-His Antibody is an anti-(H)5 mouse monoclonal for the “highly specific detection of C-terminal, N-terminal and internal His tags”. As described on the product’s web page (https://www.qiagen.com/se/products/discovery-and-translational-research/protein-purification/tagged-protein-expression-purification-detection/anti-his-antibodies-bsa-free/?catno=34660) and in the QIAexpress® Detection and Assay Handbook (Fourth Edition/July 2015) that can be downloaded from the same URL, this antibody recognizes its epitope with nanomolar affinity, can detect ~50 pg protein in Western blots (using a chemiluminescent substrate) and has been validated against many different proteins. We have abundantly used it in our previous work (see for example PMID 26850170), and repeatedly validated it by also using it to probe, as negative controls, conditioned media samples from cells that that do not express His-tagged protein. |

## Eukaryotic cell lines

Policy information about cell lines

| | |
|---|---|
| Cell line source(s) | HEK293T: laboratory of Prof. A. Radu Aricescu (University of Oxford, UK; now at the MRC Laboratory of Molecular Biology, Cambridge, UK) (PMID 3031469); the commercial source for this cell line was ATCC cat. no. CRL-3216, RRID CVCL_0063.<br>Expi293F GnTI-: Thermo Fisher Scientific cat. no. A39240. |
| Authentication | Cell line authentication was performed by the commercial sources described above, which guarantee their authenticity; no additional authentication was performed by either Prof. Aricescu or our laboratory. However, even though we did not verify cell line identities genetically, the results reported in this manuscript and other work in the laboratory showed that the type of glycosylation of the recombinant proteins expressed in these cell lines was consistent with their expected genetic background. Namely, enzymatic deglycosylation and/or mass spectrometric analysis showed that the glycans attached to recombinant proteins expressed in HEK293T were mostly complex-type (except in notable cases such as UMOD N275 (Fig. 2c) and GP2 N65 (Extended Data Fig. 8)), whereas those attached to proteins expressed in Expi293F GnTI- cells were high-mannose-type. |
| Mycoplasma contamination | Each cell line was tested for mycoplasma contamination by the respective source. We confirmed that the HEK293T cell line was mycoplasma-free by using a PCR Mycoplasma Test Kit II (Applichem cat. no. A8994). |
| Commonly misidentified lines<br>(See ICLAC register) | No commonly misidentified cell lines were used. |

## Human research participants

Policy information about studies involving human research participants

| | |
|---|---|
| Population characteristics | The research participant is a healthy male, who was 49 year old at the time of sample collection. |
| Recruitment | The participant is one of the authors of the manuscript (L.J.), who received no compensation. |
| Ethics oversight | No ethical approval was deemed necessary by the participant's department (Karolinska Institutet, Department of Biosciences and Nutrition), as he used his own urine. |

Note that full information on the approval of the study protocol must also be provided in the manuscript.
