# Supplementary figures and images for: Structure of the decoy module of human glycoprotein 2 and uromodulin and its interaction with bacterial adhesin FimH
Source: Nat Struct Mol Biol. 2022 Mar 10;29(3):190–3. doi: 10.1038/s41594-022-00729-3 (PMC8930769; doi:10.1038/s41594-022-00729-3)

**Fig. 1b**

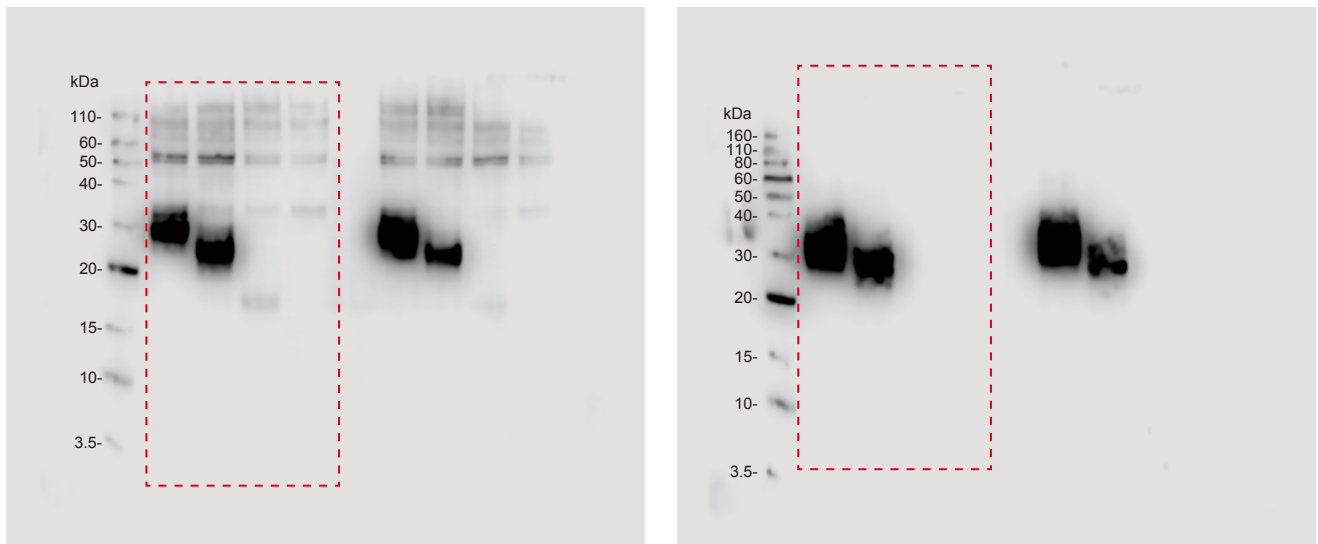

Supplement: Source Data Fig. 1 — Unprocessed western blots. [file 41594_2022_729_MOESM4_ESM.pdf]

**Fig. 2c**

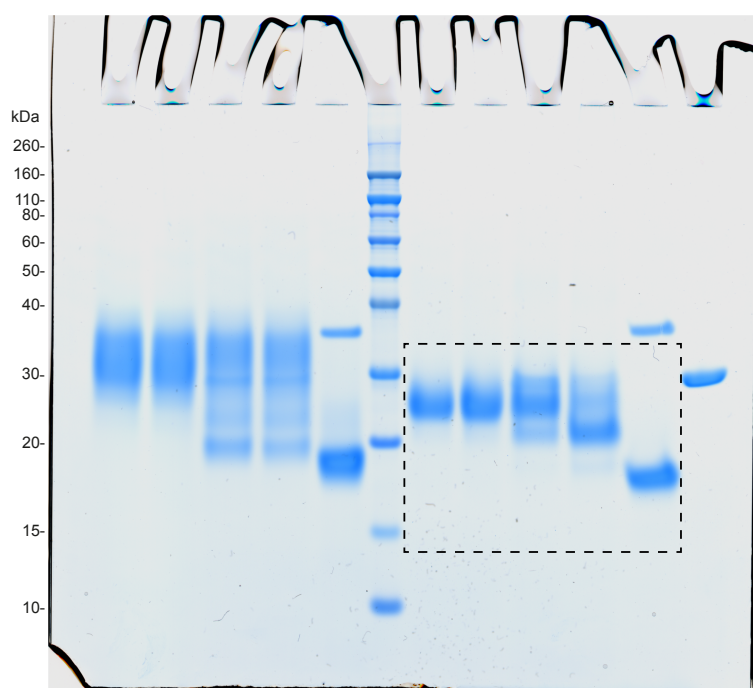

Supplement: Source Data Fig. 2 — Unprocessed gel. [file 41594_2022_729_MOESM5_ESM.pdf]

**Extended Data Fig. 2a-b**

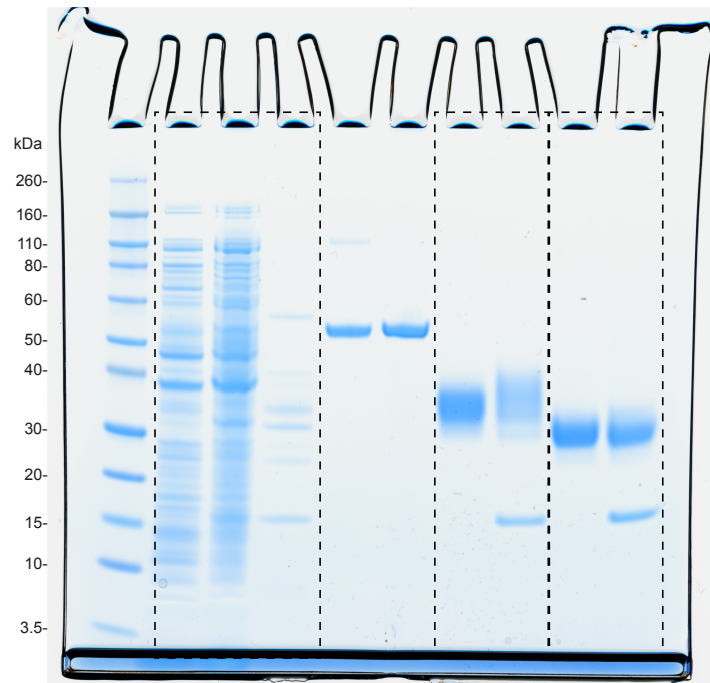

**Extended Data Fig. 2b**

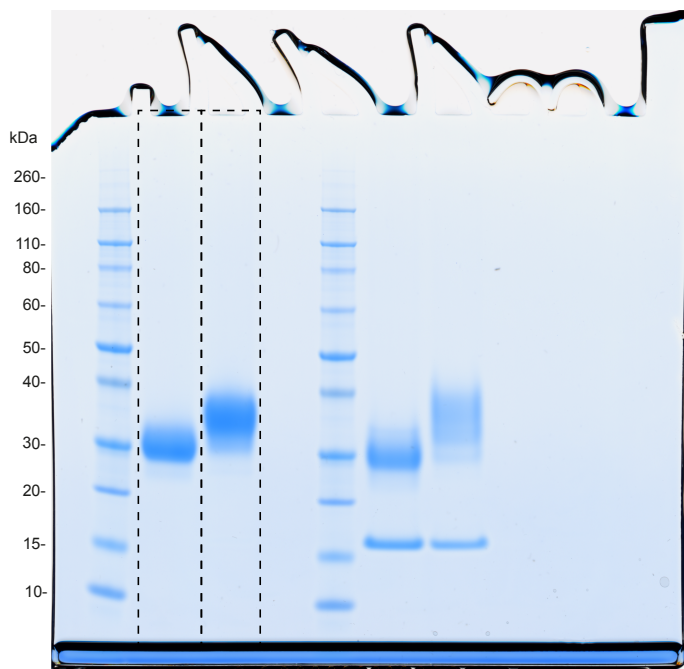

**Extended Data Fig. 2c**

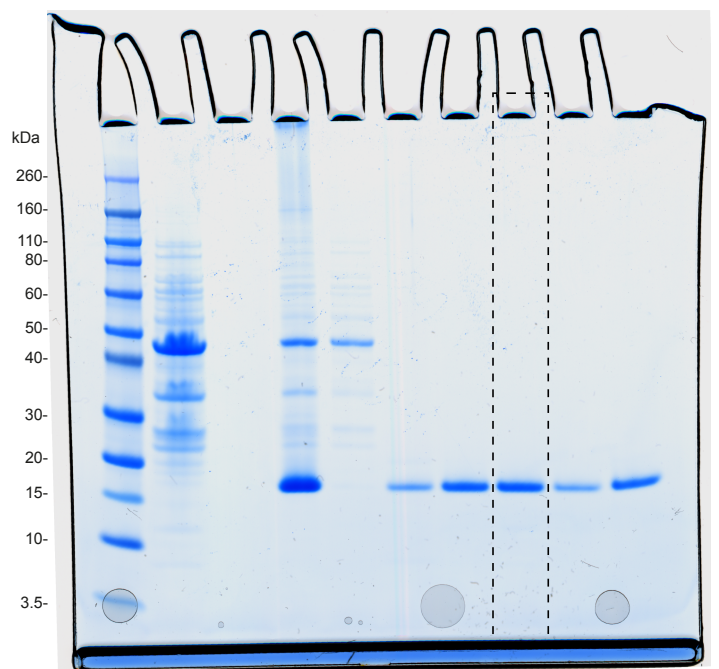

Supplement: Source Data Extended Data Fig. 2 — Unprocessed gels. [file 41594_2022_729_MOESM6_ESM.pdf]

Extended Data Fig. 7b

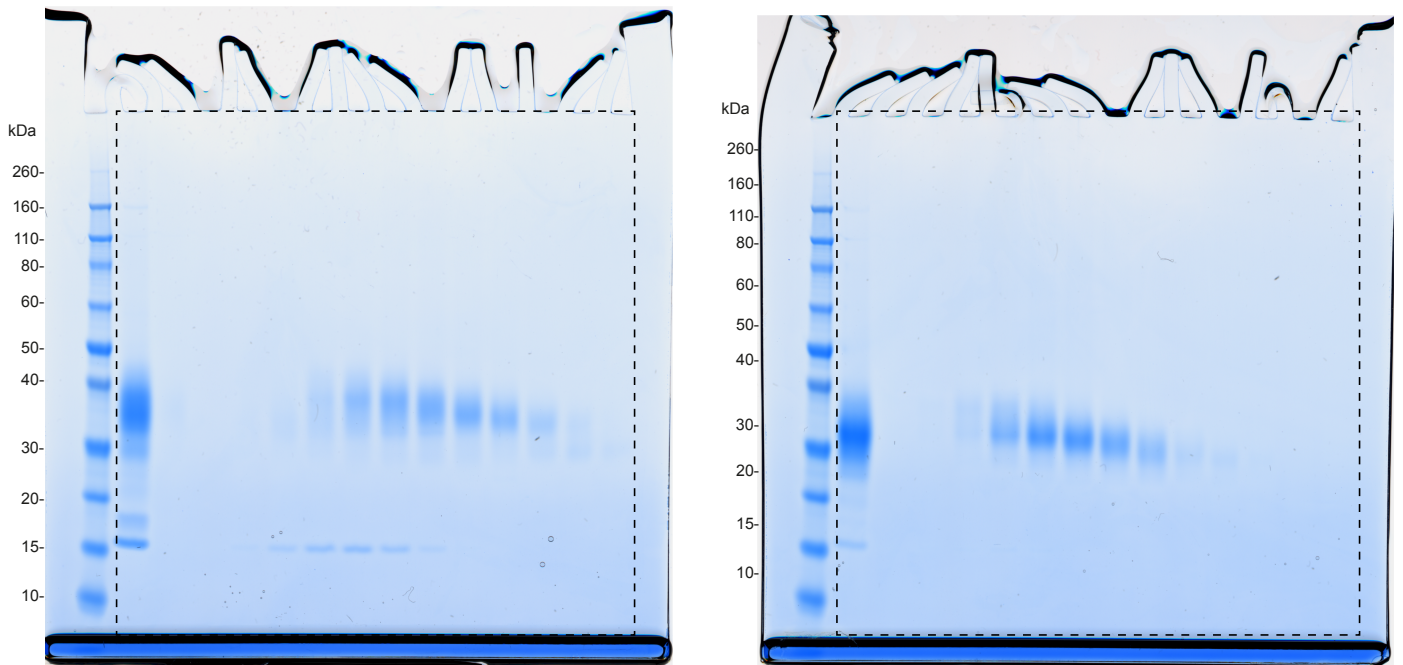

Supplement: Source Data Extended Data Fig. 7 — Unprocessed gels. [file 41594_2022_729_MOESM7_ESM.pdf]

**Extended Data Fig. 9**

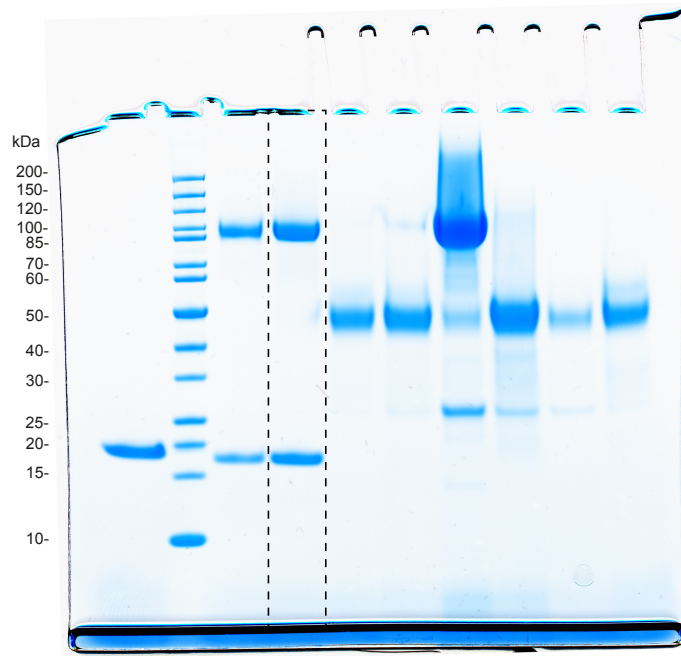

Supplement: Source Data Extended Data Fig. 9 — Unprocessed gel. [file 41594_2022_729_MOESM8_ESM.pdf]
